# Evaluation of stirring time through a rumen simulation technique: Influences on rumen fermentation and bacterial community

**DOI:** 10.3389/fmicb.2023.1103222

**Published:** 2023-03-03

**Authors:** Zuo Wang, Quan Li, Xinyi Lan, Weijun Shen, Fachun Wan, Jianhua He, Shaoxun Tang, Zhiliang Tan

**Affiliations:** ^1^College of Animal Science and Technology, Hunan Agricultural University, Changsha, Hunan, China; ^2^Key Laboratory of Agro-Ecological Processes in Subtropical Region, National Engineering Laboratory for Pollution Control and Waste Utilization in Livestock and Poultry Production, Hunan Provincial Key Laboratory of Animal Nutritional Physiology and Metabolic Process, Institute of Subtropical Agriculture, Chinese Academy of Sciences, Changsha, Hunan, China

**Keywords:** rumen simulation technique, stirring time, rumen fermentation, ruminal microbiota, full-length 16S rRNA gene sequencing

## Abstract

**Introduction:**

Rumen motility is a key element that influences ruminant nutrition, whereas little is known about the effects of rumen contraction duration on rumen fermentation and ruminal microbiome. We previously reported that proper rotation speed of a rumen simulation technique (RUSITEC) system enhanced rumen fermentation and microbial protein (MCP) production. In the present study, different contraction durations and intervals were simulated by setting different stirring times and intervals of the stirrers in a RUSITEC system. The objective of this trial was to evaluate the influences of stirring time on rumen fermentation characteristics, nutrient degradation, and ruminal bacterial microbiota *in vitro*.

**Methods:**

This experiment was performed in a 3 × 3 Latin square design, with each experimental period comprising 4 d for adjustment and 3 d for sample collection. Three stirring time treatments were set: the constant stir (CS), the intermittent stir 1 (each stir for 5 min with an interval of 2 min, IS1), and the intermittent stir 2 (each stir for 4 min with an interval of 3 min, IS2).

**Results:**

The total volatile fatty acid (TVFA) concentration, valerate molar proportion, ammonia nitrogen level, MCP density, protozoa count, disappearance rates of dry matter, organic matter, crude protein, neutral detergent fiber, and acid detergent fiber, emissions of total gas and methane, and the richness index Chao 1 for the bacterial community were higher (*p* < 0.05) in the IS1 when compared to those in the CS. The greatest TVFA, MCP, protozoa count, nutrient disappearance rates, gas productions, and bacterial richness indices of Ace and Chao 1 amongst all treatments were observed in the IS2. The relative abundance of the genus *Treponema* was enriched (*p* < 0.05) in CS, while the enrichment (*p* < 0.05) of *Agathobacter ruminis* and another two less known bacterial genera were identified in IS2.

**Discussion:**

It could be concluded that the proper reduction in the stirring time might help to enhance the feed fermentation, MCP synthesis, gas production, and the relative abundances of specific bacterial taxa.

## Introduction

The motility of the rumen plays a vital role in ruminant nutrient degradation, and could be featured by cyclic movements basically including the primary contraction and the secondary contraction (Ruckebusch, 1988; Beauchemin, 2018). The primary contraction basically facilitates the mix of rumen ingesta and microbial fermentation, as well as the transfer of rumen digesta into the omasum, whereas the secondary contraction usually leads to the eructation of ruminants (Imran et al., 2011; Arai et al., 2019). It was observed by the transabdominal ultrasonography on cows that, the average time interval between the two successive peaks of ruminal contractions was 48.6 ± 1.53 s, without the differentiation between the primary and secondary ruminal contractions (Imran et al., 2011). Through the ultrasonographic examinations performed on dairy cows, Braun and Schweizer (2015) found that the duration of a complete cycle of the primary contraction was approximately 45 s. As significant variables of rumen motility, the duration and interval of ruminal contractions could be bound up with the production performance, health, and welfare of ruminants (Braun and Schweizer, 2015; Arai et al., 2019). However, until now, reports on the exact time of the contraction duration and interval in different ruminants, and their impacts on the rumen fermentation and ruminal microbiota are rather limited.

With the constant inflows and outflows, the rumen simulation technique (RUSITEC) system could serve as a relatively more effective tool in assessing rumen fermentation *in vitro* in comparison with the traditional method of batch culture (Deitmers et al., 2022). So far, two major types of the RUSITEC system have been separately invented and are commonly applied: the semi-continuous culture system and the continuous culture system (Hoover et al., 1976; Czerkawski and Breckenridge, 1977). These artificial rumen systems vary from simple vessel sorts with different volumes to complex continuous-flow types with diverse stirrer, and various in- and outflow systems (Deitmers et al., 2022). In our previous investigation on the impacts of rotation speed on rumen fermentation and ruminal microorganisms, a novel dual-flow continuous RUSITEC system was developed and adopted with detailed descriptions (Adebayo Arowolo et al., 2022). This dual-flow continuous RUSITEC system can also be used to examine our hypothesis that the contraction duration and interval could affect the rumen microbial fermentation, through the simulation of different contraction durations and intervals by setting different stirring times and intervals for the stirrer and the elimination of the interferences from other irrelevant factors.

Therefore, the aim of the current study was to evaluate the influences of stirring time on nutrient degradation, fermentation characteristics, and bacterial microflora in a RUSITEC system. We assume that this study could not only gain more insights into the biofunction of rumen motility pattern, but also help to improve the validity of RUSITEC.

## Materials and methods

### Animals, diets, and management

All animal procedures in the present study were reviewed and approved by the Animal Care Committee (approval number: 20211003), College of Animal Science and Technology, Hunan Agricultural University, Changsha, China. Four rumen-fistulated Xiangxi yellow cattle (*Bos taurus*) with the body weight of 271 ± 28.7 kg (mean ± SD) were used and fed a basal total mixed ration (TMR; Table 1) in this experiment. All cattle were housed in a tie-stall barn and fed *ad libitum* twice per day at 08:00 and 20:00 h with free access to fresh water.

**Table 1:** Components and chemical composition of the basal TMR ration.

Component, g/kg DM	Chemical composition, g/kg DM		
Oat hay	600	OM2	921
Unhusked rice	112	CP^3^	100.8
Corn meal	100	NDF^4^	560.7
Soybean meal	34.8	ADF^5^	378.6
Wheat bran	56	EE^6^	37.2
Rice bran	28	Ash	79
Shotcrete corn husk	8	Ca	6.1
Soybean malt powder	12	P	3.5
Brown rice	12		
Soybean hull	8		
Rumen-protected fat powder	1.2		
Unite bran	5		
Expanded Urea	3.2		
Premix^1^	19.8		

^1^Every 1 kg of premix contained 260 mg of Cu, 3,200 mg of Fe, 160 mg of Mn, 10 mg of I, 3 mg of Co, 3 mg of Se, 20,000 IU of vitamin A, 800 IU of vitamin D3, and 75 IU of vitamin E; ^2^OM, organic matter; ^3^CP, crude protein; ^4^NDF, neutral detergent fiber; ^5^ADF, acid detergent fiber; ^6^EE, ether extract.

### Rumen simulation technique fermentation

The procedures for the construction and operation of the RUSITEC system adopted in this experiment were previously described by Adebayo Arowolo et al. (2022); Supplementary Figure S1. Before the morning feeding, rumen contents were collected from 4 rumen-cannulated cattle and pooled, and further strained through 4 layers of cheesecloth under a continuous CO_2_ stream. Subsequently, the strained rumen liquid was mixed with the prewarmed McDougall’s buffer (McDougall, 1948) at the ratio of 1:1 under a constant stream of CO_2_. The basal TMR diet fed to the donor cattle was also used as the substrate for the RUSITEC incubation. Twenty grams DM of the TMR feed was introduced into each fermentation vessel, together with 1,000 mL of buffered rumen liquid under a continuous CO_2_ stream. Through circulating hot water inside the water jacket, the temperature of each vessel was kept at 39°C. All the 6 fermentation vessels were randomly assigned to 3 different stirring time treatments: the constant stir (CS), the intermittent stir 1 (each stir for 5 min with an interval of 2 min, IS1), and the intermittent stir 2 (each stir for 4 min with an interval of 3 min, IS2), with 2 vessels for each stirring time treatment. The different stirring times and intervals were set based on our previous observation on the reticuloruminal contractions in goats (unpublished). The present experiment was performed in a replicated 3 × 3 Latin square design, and every fermentation vessel was successively allocated to each of the 3 stirring frequencies through 3 experimental periods. Every experimental period lasted for 7 days, consisting of 4 days for adjustment and 3 days for sample collection. The rotation speed of the stirrer was set at 26 rpm. To achieve the daily fluid turnover of the RUSITEC fermentation system, the passage rate was maintained at 0.06 / h by infusing the McDougall’s buffer into the vessels through the injection pump. Twenty grams of the TMR feed was weighed into each fermentation vessel twice daily at 08:00 and 20:00 h, during which nitrogen gas was injected to prevent the intrusion of ambient air.

### Sample collection

The sample collection regarding the RUSITEC system in the current experiment was performed by mainly referring to the protocols of our prior study (Adebayo Arowolo et al., 2022) with slight modifications. In brief, the gas emitted from each fermentation vessel was introduced into the associated gas bag with its volume measured, and approximately 20 ml of gas samples were collected from the bags before morning feeding on days 5, 6, and 7 for later analysis of methane (CH_4_) concentrations. Liquid samples were collected prior to morning feeding on days 5–7 *via* the overflow ports, with pH measured using pH meter (PHS-3C, INESA 123 Scientific Instrument Co., Ltd., Shanghai, China). Subsequently, samples were later analyzed for ammonia nitrogen (NH_3_-N), volatile fatty acids (VFAs), and microbial protein (MCP) determination using the methods described by (Adebayo Arowolo et al. (2022). For the bacterial microflora analysis, samples collected on days 5–7 from each vessel were firstly evenly combined, and then the combined samples from the two vessels with the same stirring time were further pooled. Solid samples were obtained *via* the discharge outlets from days 5 to 7, for the assessment of nutrient disappearance. All samples were immediately frozen in liquid nitrogen and preserved at −80°C before subsequent analysis.

### Chemical and biochemical analysis

Dry matter (DM; method 930.15), ash (method 942.05), crude protein (CP; method 2001.11), ether extract (EE, method 920.39), neutral detergent fiber (NDF; method 2002.04), and acid detergent fiber (ADF; method 973.18) in the TMR feed and fermentation solid samples were analyzed according to the instructions of AOAC (2005). The contents of calcium (Ca) and phosphorus (P) in the basal diet were determined as previously depicted (Tang et al., 2019). The measurements for the NH_3_-N and VFA in the rumen fluid were performed using our previous methods (Wang et al., 2016, 2021). The analysis for MCP in the rumen liquid samples and CH_4_ in the gas samples were carried out in accordance with the prior procedures (Adebayo Arowolo et al., 2022). The protozoa in the rumen fluid were counted microscopically, following the methods described by Williams and Coleman (1992) and Kišidayová et al. (2021).

### Full-length 16S rRNA gene sequencing and bioinformatic analysis

For the full-length 16S rRNA gene sequencing and relevant bioinformatic analysis of the bacterial microbiome in the rumen fluid samples, the major processes including genomic DNA isolation, PCR amplification, amplicon sequencing, raw sequencing data pretreatment, operational taxonomic unit (OTU) taxonomic classification, Alpha diversity and Beta diversity analysis, and Tax4Fun function prediction were successively performed by exactly following the approaches depicted in our prior investigation (Wang et al., 2021). All the raw sequences acquired from the sequencing were deposited to the sequence read archive (SRA) of the NCBI database under the accession number PRJNA877046.

### Statistical analysis

To assess the effects of stirring time on RUSITEC fermentation parameters, nutrient disappearance rates, and Alpha diversity indexes, the corresponding data were analyzed using the GLM procedure of SPSS statistics (V23.0, IBM Corporation, Armonk, United States). The following model was used for the analysis of data:

Y_ijklm_ = μ + P_i_ + V_j(l)_ + T_k_ + S_l_ + H_m_ + H_Tmk_ + S_Tlk_ + E_ijkl_.

where Y_ijklm_ is the dependent variable, μ is the overall mean, P_i_ is the effect of period i, V_j(l)_ is the effect of vessel j within square l, T_k_ is the effect of stirring time treatment k, S_l_ is the square effect (l = 1 or 2), H_m_ is the effect of sampling date m, H_Tmk_ is the interaction between sample time m and treatment k, S_Tlk_ is the interaction between square l and treatment k, and E_ijklm_ is the random residual error. Sampling date was considered as the repeated measurement. The observed means were compared by Duncan’s multiple range test. Least squares means were presented throughout the text. Statistical difference was, respectively, considered as significant or highly significant at *p* < 0.05 or *p* < 0.01, while trend was discussed at 0.05 < *p* ≤ 0.10. Linear discriminant analysis effect size (LEfSe) was employed to compare relative abundances of microbial taxa within treatments, and significant differences were declared by a linear discriminant analysis (LDA) score > 3.0 and *p* < 0.05.

## Results

### Rumen simulation technique fermentation characteristics

The pH of the rumen fluid decreased (*p* < 0.01) with the increase in time interval between stirring, while an increase (*p* < 0.01) in total volatile fatty acid (TVFA) concentration was observed in response to the increasing time interval (Table 2). The molar proportions of valerate in both IS1 and IS2 were higher (*p* < 0.01) than that in the CS group. The higher (*p* < 0.01) concentrations of MCP, accompanied with the lower (*p* < 0.01) concentrations of NH_3_-N in IS1 and IS2 were found when compared to CS. In addition, the number of protozoa in the rumen liquid increased (*p* < 0.01) and peaked in IS2.

**Table 2:** Influences of different stirring times on RUSITEC fermentation characteristics.

Item	Treatment			SEM^4^	*P*-value
	CS^1^	IS1^2^	IS2^3^		
pH	6.97^a^	6.69^b^	6.57^c^	0.187	<0.01
TVFA^5^ (mmol/L)	52.15^c^	60.35^b^	62.99^a^	0.839	<0.01
VFA profile (mol/100 mol)					
Acetate	72.49	72.77	72.94	1.668	0.811
Propionate	18.58	18.22	18.01	1.449	0.631
Butyrate	3.61	3.57	3.58	0.252	0.966
Isobutyrate	1.95	1.89	1.89	0.117	0.359
Valerate	1.39^b^	1.64^a^	1.69^a^	0.146	<0.01
Isovalerate	1.97	1.89	1.89	0.346	0.817
A:P^6^	3.93	4.03	4.08	0.665	0.644
NH_3_-N (mmol/L)	6.29^a^	5.44^b^	5.09^c^	0.108	<0.01
MCP^7^ (mg/mL)	6.83^c^	11.44^b^	13.26^a^	4.617	<0.01
Protozoa count (10^4^/mL)	0.92^c^	1.19^b^	1.28^a^	0.045	<0.01

^a,b,c^Means within a row for treatments that do not have a common superscript differ. ^1^CS, constant stir; ^2^IS1, intermittent stir 1; ^3^IS2, intermittent stir 2; ^4^SEM, standard error of means for treatments; ^5^TVFA, total volatile fatty acid; ^6^A:P, the ratio of acetate to propionate; ^7^MCP, microbial protein.

### Nutrient disappearances and gas emissions

The disappearance of DM, OM, and CP increased (*p* < 0.01) as the time interval increased, and the disappearance of NDF and ADF in IS1 and IS2 were higher (*p* < 0.01) than the CS treatment (Table 3). Total gas and CH_4_ production increased (*p* < 0.01) with the increasing time interval, with the highest values observed in IS2.

**Table 3:** Influences of different stirring times on nutrient disappearance rates and gas emissions.

Item	Treatment			SEM^4^	*P*-value
	CS^1^	IS1^2^	IS2^3^		
Nutrient disappearance (%)					
DMD^5^	66.87^c^	72.67^b^	73.83^a^	0.343	<0.01
OMD^6^	68.07^c^	70.73^b^	72.16^a^	0.381	<0.01
CPD^7^	63.81^c^	66.69^b^	69.64^a^	0.350	<0.01
NDFD^8^	55.93^b^	63.74^a^	64.33^a^	0.475	<0.01
ADFD^9^	27.75^b^	30.75^a^	30.58^a^	0.161	<0.01
Gas production					
Total gas (L/d)	1.44^c^	1.64^b^	1.86^a^	0.030	<0.01
CH_4_ (mL/d)	223^c^	278^b^	340^a^	8.2	<0.01

^a,b,c^Means within a row for treatments that do not have a common superscript differ. ^1^CS, constant stir; ^2^IS1, intermittent stir 1; ^3^IS2, intermittent stir 2; ^4^SEM, standard error of means for treatments; ^5^DMD, dry matter disappearance; ^6^OMD, organic matter disappearance; ^7^CPD, crude protein disappearance; ^8^NDFD, neutral detergent fiber disappearance; ^9^ADFD, acid detergent fiber disappearance.

### Taxonomic annotation for the ruminal bacterial microflora

During the bioinformatic analysis, the average numbers of the filtered CCS sequences and the OTUs obtained per sample were 4,787 ± 610 and 124 ± 13, respectively (Supplementary Table S1). A total of 8 bacterial phyla were annotated across all the samples (Supplementary Table S2), with Proteobacteria (45.6 ± 9.89%), Bacteroidota (43.0 ± 7.64%), and Firmicutes (9.0 ± 3.73%) jointly taking up 97.5 ± 1.33% of the entire bacterial community (Supplementary Figure S2). As to the bacterial genera, the *Rikenellaceae_RC9_gut_group* (30.8 ± 5.53%), *Succinivibrionaceae_UCG_002* (21.5 ± 12.80%), and *Ruminobacter* (23.6 ± 11.03%) were successively detected as the 3 most predominant bacterial taxa (Supplementary Figure S3). The ruminal bacterial microbiota, at the species level, was basically dominated by the *Ruminobacter amylophilus* (23.6 ± 11.03%), *unclassified_Succinivibrionaceae_UCG_002* (21.4 ± 12.79%), and *Bacteroidales_bacterium_P13* (14.2 ± 2.77%; Supplementary Figure S4). In sum, 150, 147, and 148 OTUs were, respectively, clustered in CS, IS1, and IS2, with none of them identified as exclusive to each treatment (Supplementary Figure S5). Those common OTUs were generally assigned to the phylum of either Proteobacteria, Bacteroidota, or Firmicutes (Supplementary Table S3).

### Diversity of ruminal bacterial populations

**Figure 1:**
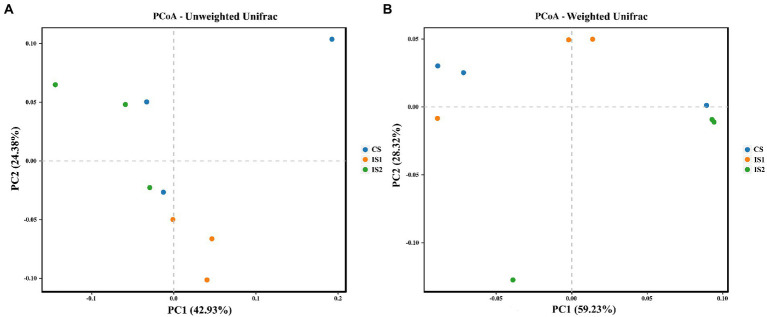
The principal coordinate analysis (PCoA) plots on the structure of the rumen bacterial populations across treatments. **(A)** PCoA based on the unweighted Unifrac matrix. **(B)** PCoA based on the weighted Unifrac matrix.

The Alpha diversity index of Ace for the bacterial microflora in the IS2 was promoted (*p* < 0.05) compared with the CS (Table 4). The Chao 1 indices in IS1 and IS2 were higher (*p* < 0.05) than the CS. As for the Beta diversity of the bacterial community, a significant treatment-dependent clustering was depicted based on the unweighted Unifrac distances (Figure 1A). According to the weighted Unifrac matrix, the bacterial population in IS2 was separate from those in CS and IS1 (Figure 1B).

**Table 4:** Influences of different stirring times on Alpha diversity indices of the bacterial population.

Item	Treatment			SEM^4^	*P*-value
	CS^1^	IS1^2^	IS2^3^		
Ace	128^b^	139^ab^	149^a^	3.3	0.010
Chao 1	127^b^	138^a^	152^a^	4.4	0.027
Shannon	4.05	4.05	4.62	0.170	0.329
Simpson	0.86	0.88	0.90	0.016	0.593

^a,b,c^Means within a row for treatments that do not have a common superscript differ. ^1^CS, constant stir; ^2^IS1, intermittent stir 1; ^3^IS2, intermittent stir 2; ^4^SEM, standard error of means for treatments.

### Differential ruminal bacterial taxa

**Figure 2:**
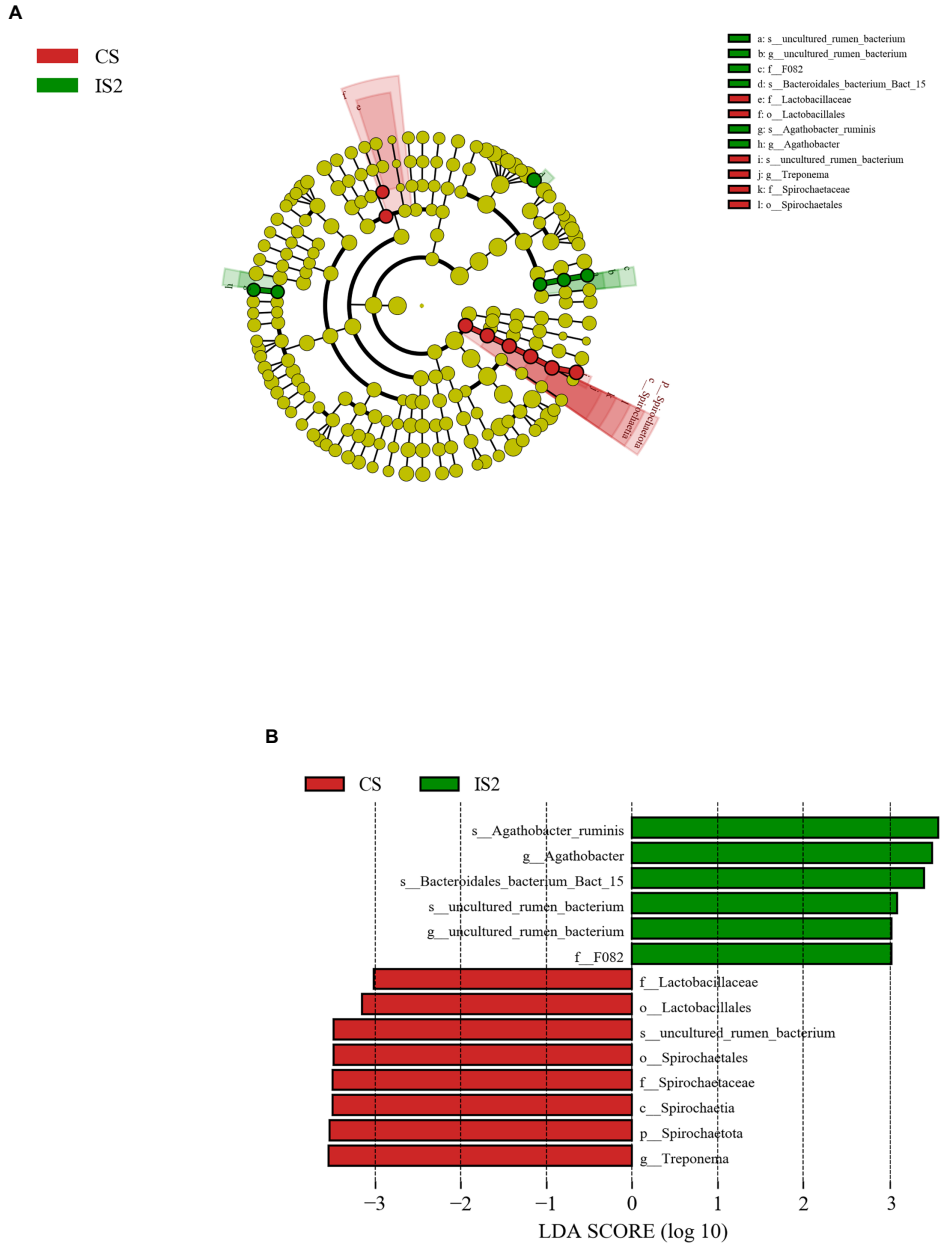
The LDA effect size (LEfSe) analysis for the differential bacterial taxa across treatments. **(A)** Cladogram displays significantly enriched bacterial taxa (from the phylum to the species level). Red: taxa abundant in the CS treatment; Green: taxa abundant in the IS2 treatment. **(B)** Bar chart displays LDA scores across treatments. The LDA scores represented the difference in relative abundance with exponent fold change of 10 across treatments. Significant differences are defined as *P* < 0.05 and LDA score > 3.0.

As was illustrated through the LEfSe analysis, the Spirochaetota, Spirochaetia, Spirochaetales, *Spirochaetaceae*, *Treponema*, and *uncultured_rumen_bacterium* were successively identified as the most differentially enriched (*p* < 0.05) bacterial taxa from the phylum to the species level in CS treatment (Figure 2). Besides, the order Lactobacillales and the affiliated family *Lactobacillaceae* were also enriched (*p* < 0.05) in CS group. In contrast, the *Agathobacter ruminis*, *Bacteroidales_bacterium_Bact_15*, and another *uncultured_rumen_bacterium* belonging to the family *F082* were the 3 bacterial species that were enriched (*p* < 0.05) in the IS2 treatment.

### Function prediction for the ruminal bacterial microbiome

**Figure 3:**
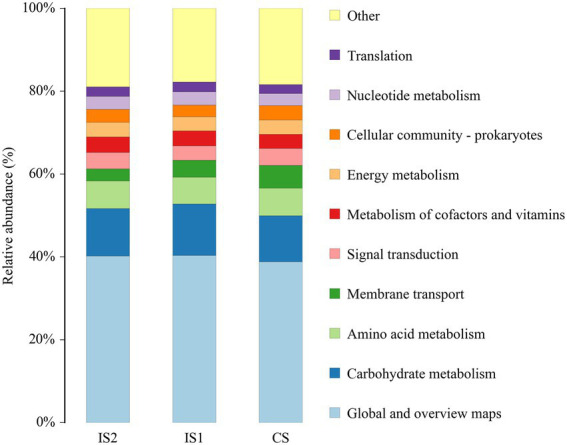
The top 10 annotated KEGG pathways (at level 2) across treatments based on Tax4Fun function prediction.

**Figure 4:**
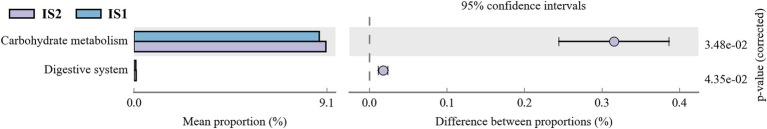
The differential KEGG pathways (at level 2) within treatments based on Tax4Fun function prediction.

According to the function prediction using Tax4Fun analysis, the global and overview maps, carbohydrate metabolism, amino acid metabolism, and membrane transport were annotated with the most dominant KEEG orthologs (KO) abundances in the top 10 KEGG pathways at level 2 (Figure 3). Moreover, it was demonstrated by the *t*-test analysis that the KEGG pathways of carbohydrate metabolism and digestive system were both enriched (*p* < 0.05) in the IS2 when compared to the IS1 (Figure 4; Supplementary Table S4).

## Discussion

The ruminal motility, which primarily consists of the primary and secondary contractions, has been proved to facilitate the microbial colonization on feed particles and thence enhance the rumen fermentation (Ruckebusch, 1988; Beauchemin, 2018). The reticulorumen contractions are crucial for the digesta mixing and movement, as well as the gas eructation (Imran et al., 2011; Arai et al., 2019). It has been detected that the strength, frequency, and duration of ruminal contractions generally grow around feeding (Egert et al., 2014; Arai et al., 2019). Conversely, declines in the rumen contractions could be a clinical sign of reticuloruminal stasis, which is usually related to metabolic disease such as ruminal acidosis, tympany, and hypocalcaemia (Arai et al., 2019). In the present study, the conversion from constant stirring to intermittent stirring significantly improved the ruminal TVFA concentration, nutrient disappearance rates, and total gas production, indicating that proper reduction of the stirring time might help to prolong the retention time during which the feed particles were degraded by the ruminal microbes in the fermentation vessels, and thereby promote the RUSITEC fermentation (Dufreneix et al., 2019; Adebayo Arowolo et al., 2022).

The NH_3_-N is exploited by the ruminal microbes in synthesizing microbial protein, and the simultaneous supply of nitrogen and energy is essential for the NH_3_-N utilization efficiency and MCP production of the ruminal microorganisms (Nocek and Russell, 1988; Bharanidharan et al., 2018; Lu et al., 2019). It has been reported that the rumen motility could help the microbes to colonize the substrate and hence contribute to the synchronization of nitrogen and energy release (Beauchemin, 2018; Adebayo Arowolo et al., 2022). In this trial, the MCP level rose significantly with the reducing stirring time, which was in line with the significant decrement in the ruminal NH_3_-N concentration. The improved MCP synthesis observed in this study further supported our previous assumption that appropriate motility could promote the acquisition of NH_3_-N by the ruminal microorganisms for MCP production (Adebayo Arowolo et al., 2022).

Our precedent investigation found that elevating the stirring speed from 5 rpm to 25 rpm quadratically increased the copy number of protozoa in the rumen solid (Adebayo Arowolo et al., 2022). In the current experiment, the highest protozoa count in the rumen fluid was exhibited in the IS2 treatment, while the protozoa number in IS2 was also higher than that in the CS group. This phenomenon could be an explanation for the significant increments of CH_4_ yield in response to the increasing interval time between stirring, as a major portion of methanogens are attached to ruminal protozoa and considered as one of the most active contributor for methanogenesis (Belanche et al., 2014; Wang et al., 2017b). It could be thus inferred that, compared to the continuous stir, reducing the stirring time and increasing the interval properly would also benefit the proliferation of rumen protozoal populations.

In the rumen liquid obtained from the RUSITEC system of this trial, the bacterial community at the phylum level was successively dominated by Proteobacteria, Bacteroidota, and Firmicutes, which further confirmed the predominance of these three bacterial phyla in the ruminal microbiome reported in precedent investigations including our prior studies (Rey et al., 2014; Wang et al., 2017a, 2020, 2021, 2022; Zhong et al., 2018). Besides, the genera *Rikenellaceae_RC9_gut_group*, *Succinivibrionaceae_UCG_002*, and *Ruminobacter*, and the species *Ruminobacter amylophilus* were, respectively, identified as the prominent ruminal bacterial taxa in this experiment, and this result was also supported by the findings of previous researches (Cui et al., 2021; Daghio et al., 2021; Wu et al., 2021).

In this study, the greatest indices of Ace and Chao 1 were both marked in IS2, implying that the intermittent stirring raised the richness of the bacterial populations when compared with the constant stirring (Granja-Salcedo et al., 2017). Since the diversity and richness of the rumen microflora are considered as the fundamental factor which could affect the rumen function (Weimer, 2015; Xie et al., 2018), the elevated richness of the bacterial microbiome might lead to the above-mentioned enhancement of rumen fermentation metrics. Further distinctions of the bacterial community within different treatments were shown in the PCoA plots based on both unweighted and weighted Unifrac matrix, which suggested the influences of stirring time on the structure of rumen bacterial microbiota.

The genus *Treponema* is a bacterial taxon that commonly detected in the rumen as a major participator involving the lignocellulose degradation (Bekele et al., 2011; Xie et al., 2018), while the *Lactobacillaceae* is a lactate-producing bacterial family precedently found in the bovine rumen (Wu et al., 2019; Baek et al., 2020). The prevalence of these two bacterial taxa in the CS treatment might indicate their preference for the continuous stirring condition, and requires further studies to be verified. Moreover, Li and Guan (2017) reported that the abundance of *Lactobacillaceae* tended to be higher in the rumen of cattle with low feed efficiency, when compared to cattle possessing high feed efficiency. It could be thereby assumed that the significant enrichment of *Lactobacillaceae* in the CS could be correlated with the lower nutrient disappearance rates than those in the IS1 and IS2. *Agathobacter ruminis* was previously isolated from the rumen of grazing sheep and cows, and it had been identified as an obligatory anaerobic and saccharolytic genus with the capacity to produce acetate and butyrate (Rosero et al., 2016). Therefore, the enrichment of *Agathobacter ruminis* might partly contribute to the boost in ruminal TVFA in IS2. Very little is known about the *Bacteroidales_bacterium_Bact_15* and *uncultured_rumen_bacterium* that were enriched in IS2. It could be presumed that these two genera might favor the environment with reduced stirring time, and more studies are necessitated to disclose their role in rumen fermentation during the frequency change.

It was noteworthy in this study that, according to the Tax4Fun analysis, the KEGG pathways of carbohydrate metabolism and digestive system were both enriched in IS2 compared with IS1. Nevertheless, no difference was observed in the abundances of metabolic pathways between IS2 and CS, despite those significant differences in fermentation parameters and nutrient disappearance rates between these two treatments. This disparity could stem from the distinctions between the bacterial populations in rumen fluid and rumen solid, differences between the metagenomics analysis and actual metabolic activities of the rumen microflora, and the limitations of Tax4Fun (Wang et al., 2017a, 2020, 2021).

## Conclusion

In conclusion, the highest TVFA, MCP, protozoa count, nutrient disappearance rates, gas production, and bacterial richness indices of Ace and Chao 1 were all presented in the IS2 treatment of this trial. In addition, the relative abundances of *Agathobacter ruminis* and another two less known bacterial genera were also enriched by raising the interval time from 0 to 3 min. The current investigation not only supported the theory that the proper cyclic contractions could be beneficial to the ruminal microbial fermentation, but also provide the improvements of RUSITEC system with more references. Further studies are required to explore the optimum stirring time and interval for the enhancement of RUSITEC fermentation, as well as to elucidate the roles and relevant mechanisms for those altered bacteria during the frequency change.

## Data availability statement

The datasets presented in this study can be found in online repositories. The names of the repository/repositories and accession number(s) can be found at: https://www.ncbi.nlm.nih.gov/, PRJNA877046.

## Ethics statement

All animal procedures in the present study were reviewed and approved by the Animal Care Committee (approval number: 20211003), College of Animal Science and Technology, Hunan Agricultural University, Changsha, China.

## Author contributions

ZW, QL, and WS designed the research. ZW, QL, XL, WS, FW, JH, ST, and ZT conducted the research. ZW and QL analyzed the data. ZW wrote the manuscript. All authors contributed to this study and read and approved the final manuscript.

## Funding

The present work was supported by the Hunan Herbivores Industry Technological System, Science and Technology Department of Xinjiang Uygur Autonomous Region (Grant No. 2022A02001-1), Hunan Provincial Science and Technology Department (Grant No. 2021RC4060), Hunan Provincial Natural Science Foundation (Grant No. 2021JJ30011), and National Natural Science Foundation of China (Grant No. 32172758).

## Conflict of interest

The authors declare that the research was conducted in the absence of any commercial or financial relationships that could be construed as a potential conflict of interest.

## Publisher’s note

All claims expressed in this article are solely those of the authors and do not necessarily represent those of their affiliated organizations, or those of the publisher, the editors and the reviewers. Any product that may be evaluated in this article, or claim that may be made by its manufacturer, is not guaranteed or endorsed by the publisher.
