# Supplementary figures and images for: Evaluation of stirring time through a rumen simulation technique: Influences on rumen fermentation and bacterial community
Source: Front Microbiol. 2023 Mar 3;14:1103222. doi: 10.3389/fmicb.2023.1103222 (PMC10026382; doi:10.3389/fmicb.2023.1103222)

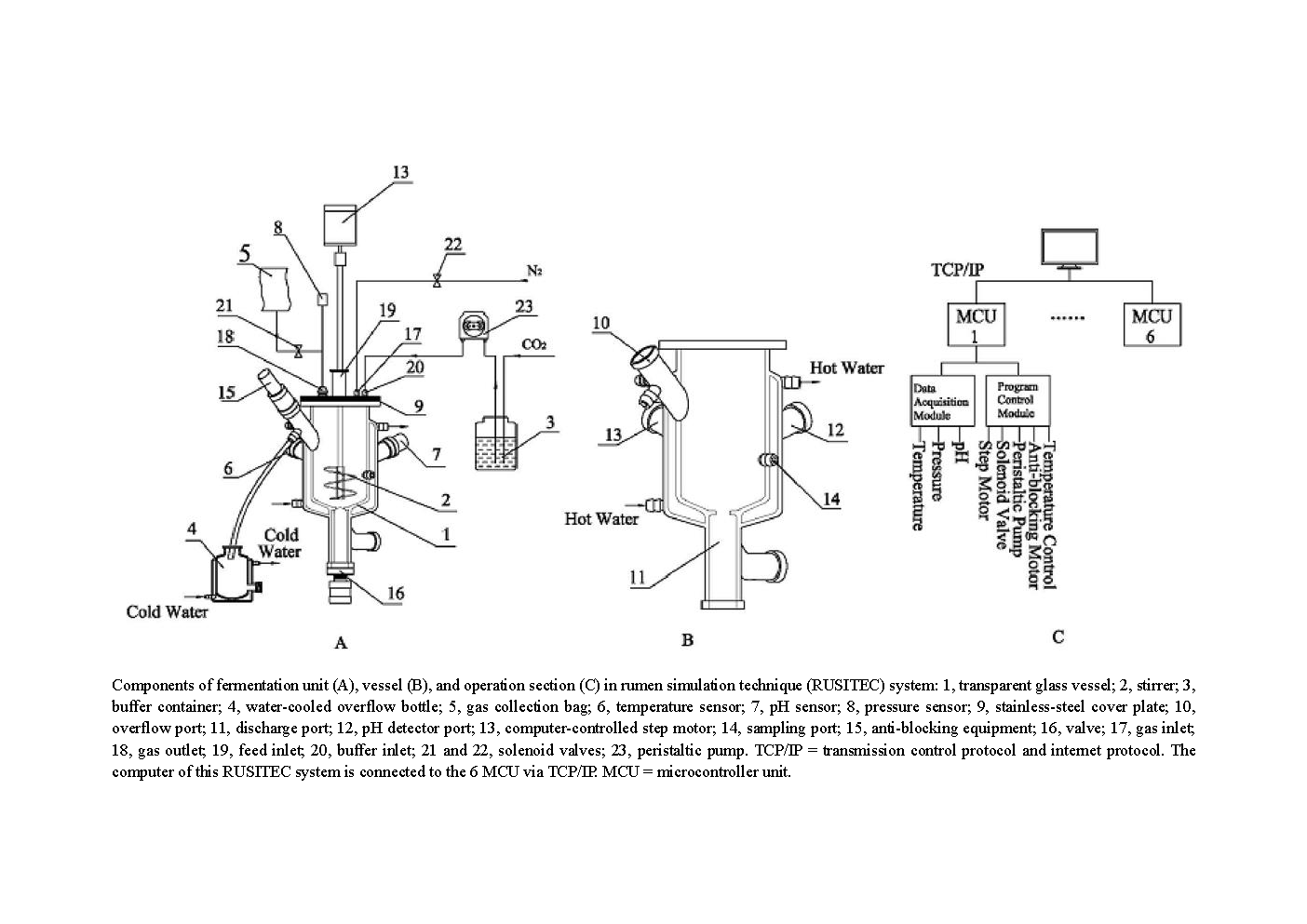

Supplement: Supplementary file 5 [file Image_1.JPEG]

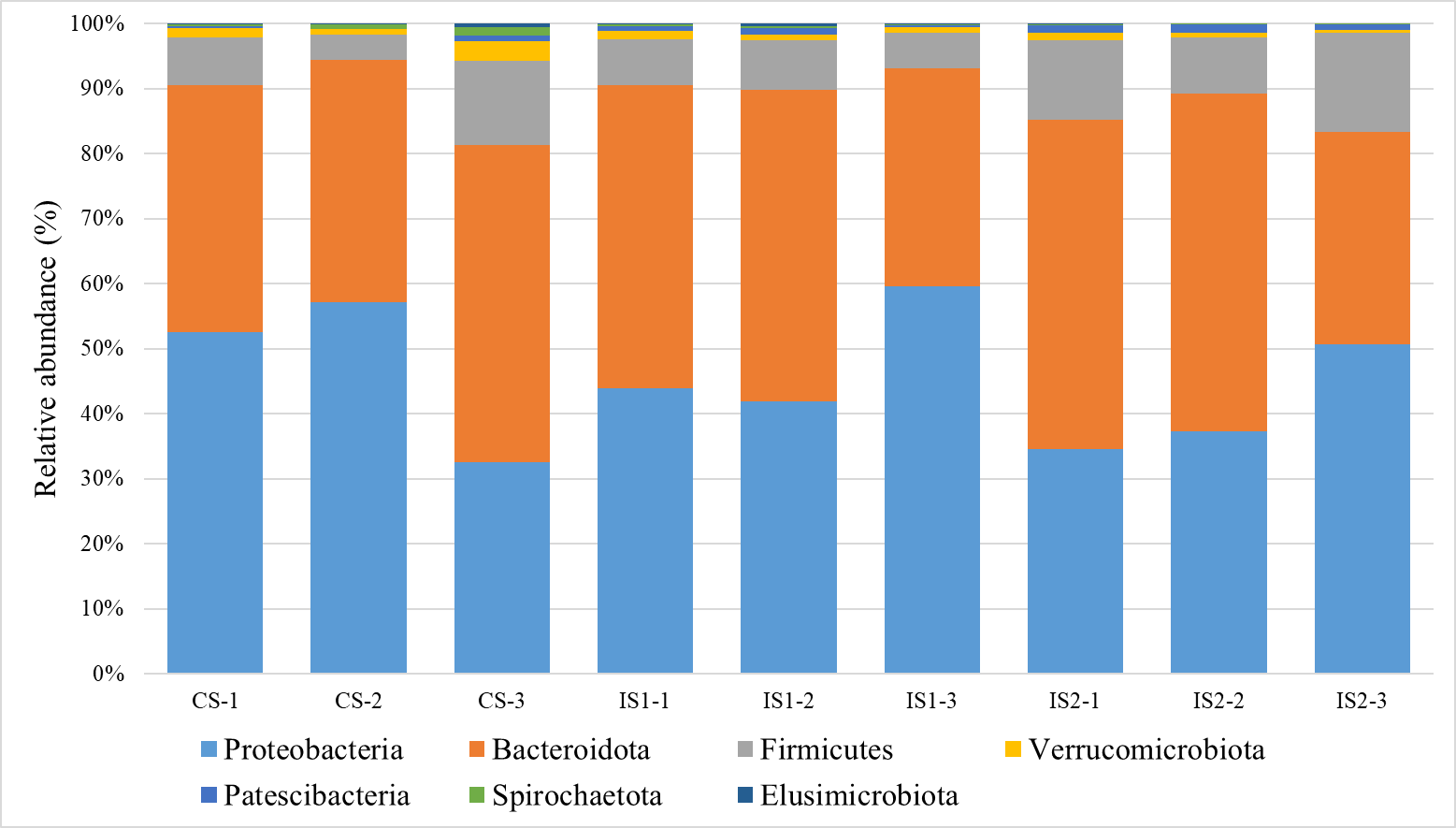

Supplement: Supplementary file 6 [file Image_2.JPEG]

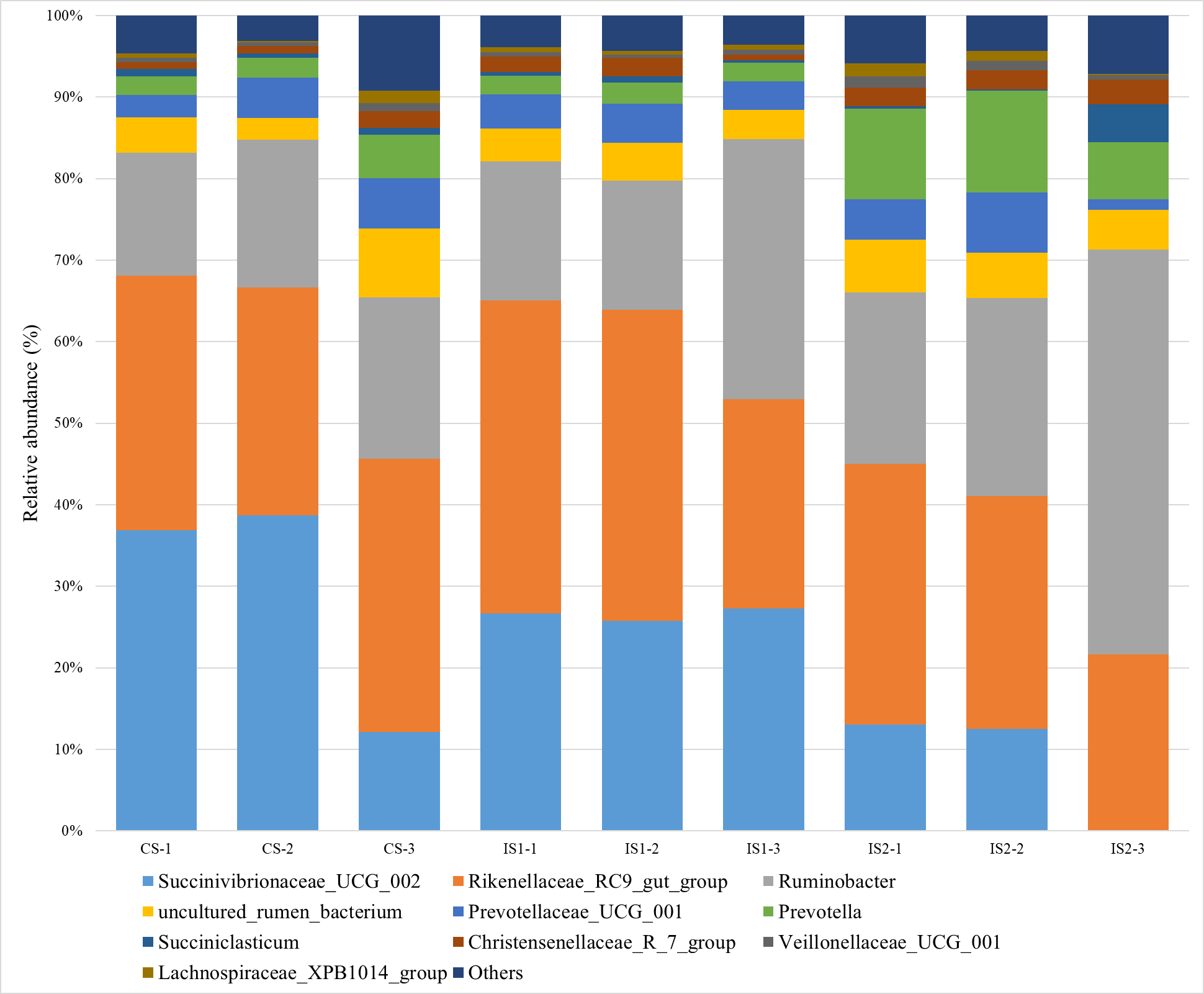

Supplement: Supplementary file 7 [file Image_3.JPEG]

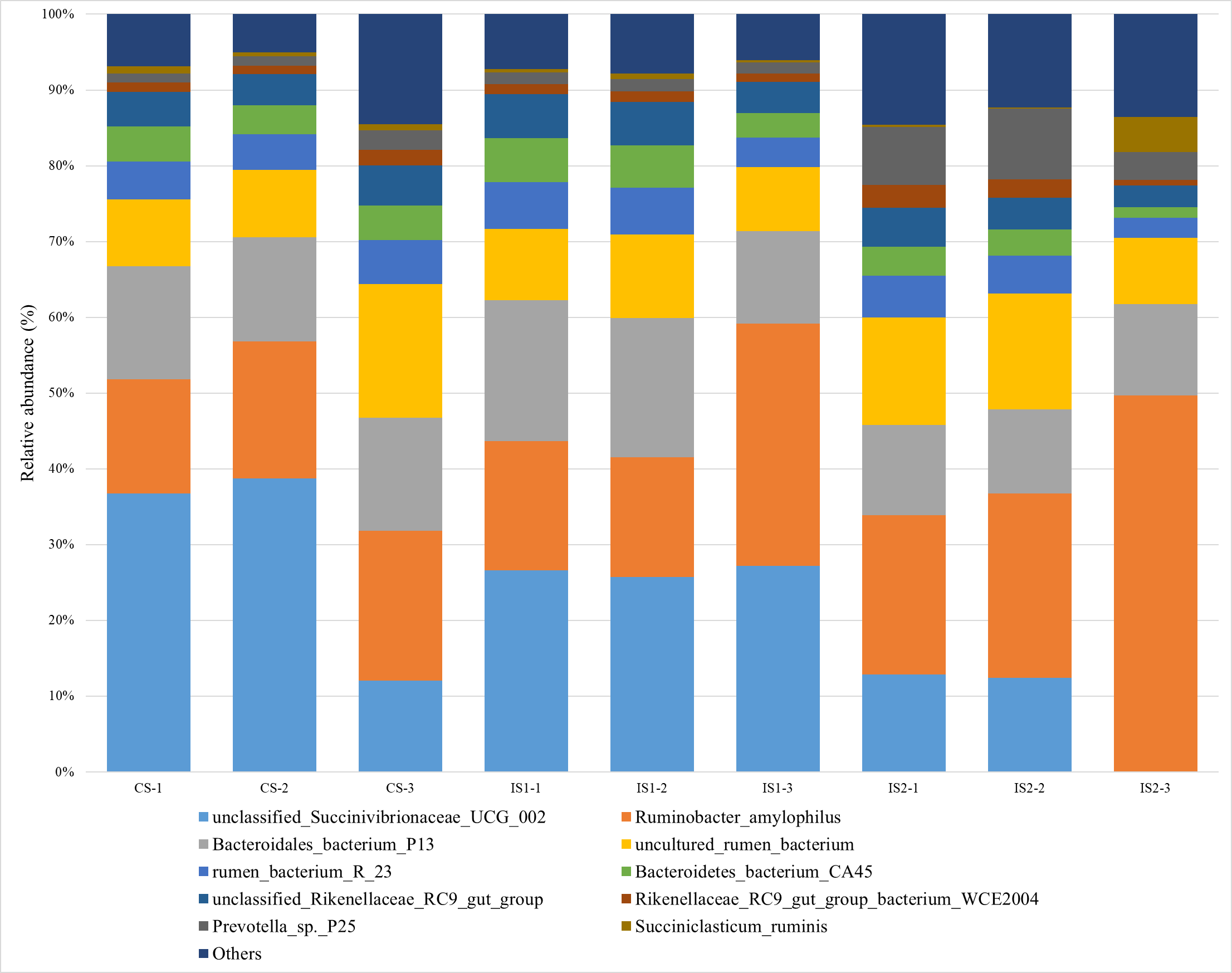

Supplement: Supplementary file 8 [file Image_4.JPEG]

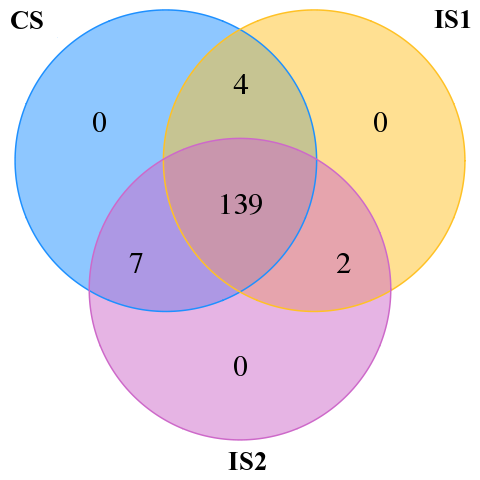

Supplement: Supplementary file 9 [file Image_5.JPEG]
